# Gastric Distention after Catheter Ablation for Atrial Fibrillation

**DOI:** 10.31662/jmaj.2025-0192

**Published:** 2025-08-08

**Authors:** Azusa Shimabukuro, Tadao Aikawa, Kenichi Iijima, Tohru Minamino

**Affiliations:** 1 Department of Cardiovascular Biology and Medicine, Juntendo University Graduate School of Medicine, Tokyo, Japan

**Keywords:** gastric distention, catheter ablation, cryoablation, atrial fibrillation

A 77-year-old man presented to the emergency department of our hospital after experiencing 5 days of anorexia and nausea. His medical history included myocardial infarction, atrial fibrillation, and lung cancer. He had undergone cryoablation for atrial fibrillation 6 days earlier and had no gastrointestinal symptoms before the procedure. His abdomen was distended but not tender; his blood pressure was 75/53 mm Hg, and his heart rate was 100 bpm. Laboratory blood tests showed an elevated white blood cell count of 14.5 × 10^9^ cells/L and a C-reactive protein level of 6.9 mg/100 mL. A chest X-ray showed pneumonia in the lower right lobe (Figure 1A); an abdominal X-ray indicated delayed gastric emptying (Figure 1B); and a computed tomography revealed gastric distention with a large amount of food residue (Figure 1C). The patient was diagnosed with catheter ablation-induced gastroparesis and aspiration pneumonia due to vomiting. His symptoms were relieved after fasting for several days and after starting mosapride citrate (15 mg/day), erythromycin (3 mg/kg three times daily) ^(1)^, and antibiotics.

**Figure 1.**
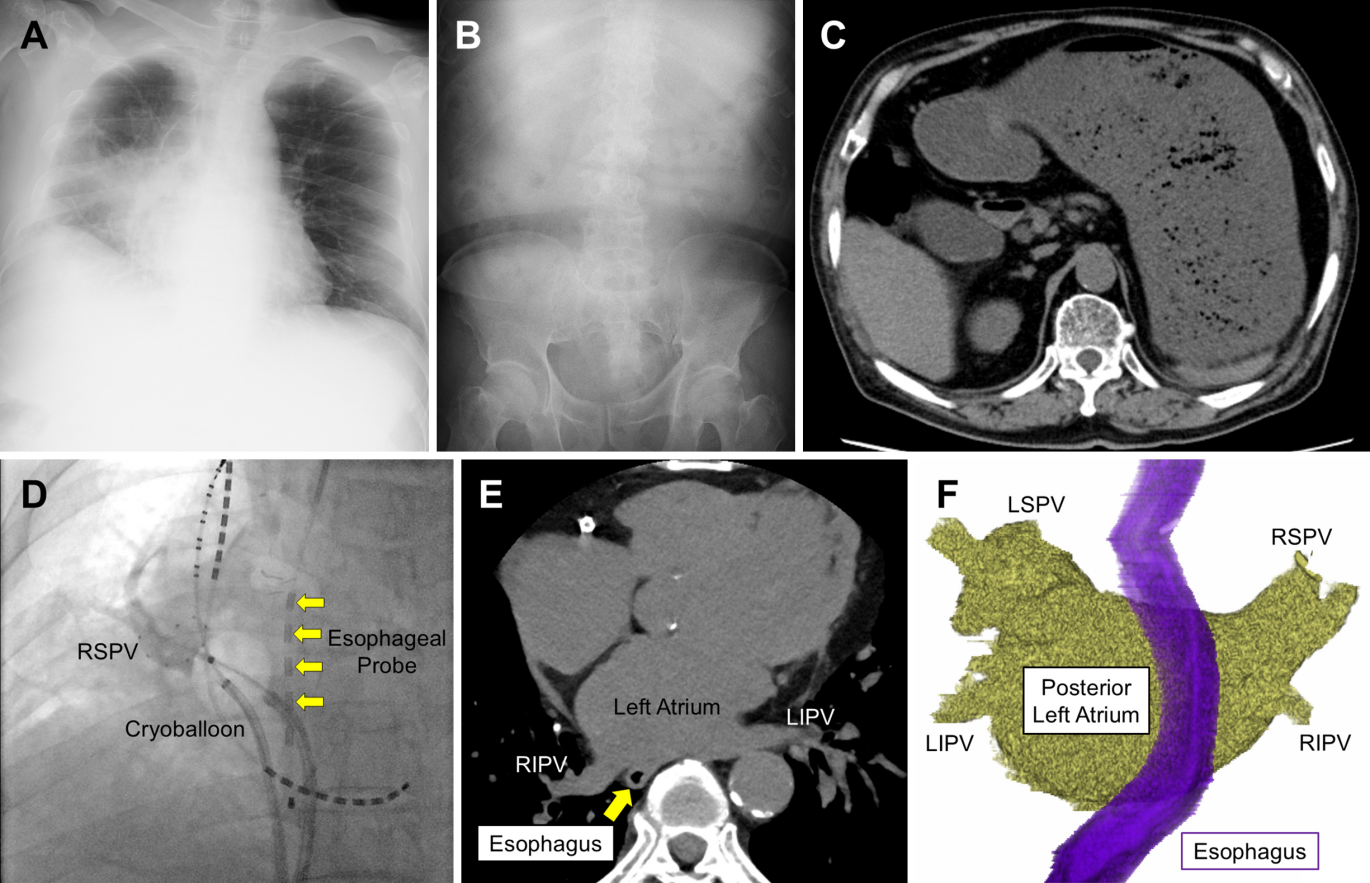
Images of gastric distention after catheter ablation for atrial fibrillation. (A-C) On admission, a chest X-ray showed pneumonia in the lower right lobe (A), an abdominal X-ray indicated delayed gastric emptying (B); and a computed tomography revealed gastric distention with a large amount of food residue (C). (D) An esophageal temperature probe (*arrows*) was used to monitor esophageal temperature during cryoablation procedures to prevent injury to the esophagus. (E, F) Non-contrast electrocardiogram-gated computed tomography images taken before the ablation showed that the esophagus was adjacent to the center of the posterior wall of the left atrium. LIPV: left inferior pulmonary vein; LSPV: left superior pulmonary vein; RIPV: right inferior pulmonary vein; RSPV: right superior pulmonary vein.

The most common mechanism of catheter ablation-induced gastroparesis is reversible injury to the periesophageal vagal nerve ^(2), (3)^. Electrical pulmonary vein isolation from the left atrium was performed using the POLARx cryoablation system (Boston Scientific). An esophageal temperature probe (Esophaster, Japan Lifeline Co., Ltd.) was used to monitor esophageal temperature during the procedure to prevent injury to the esophagus (Figure 1D, arrows). Freezing was terminated if the balloon temperature reached −70°C or the esophageal temperature reached 15°C. The freezing time duration for each pulmonary vein ranged from 150 to 180 seconds, and all veins were successfully isolated with a single freezing cycle. In this case, the esophageal temperature never fell below 15°C, and no additional ablation was performed on areas of the left atrium other than the pulmonary veins. Non-contrast electrocardiogram-gated computed tomography images taken before the ablation showed that the esophagus was adjacent to the center of the posterior wall of the left atrium (Figure 1E and F). This location has been reported to increase the risk of catheter ablation-induced gastroparesis ^(4)^.

Catheter ablation-induced gastroparesis is a rare complication with an incidence rate of 0.23% ^(5)^, which is not well recognized by general practitioners and may be masked by a delayed onset after catheter ablation. Pulmonary vein isolation is widely recognized as an effective treatment for atrial fibrillation, and the number of procedures performed increases yearly. Therefore, clinicians should be vigilant for signs of delayed gastric emptying and the risk of aspiration after ablation procedures involving regions adjacent to the vagus nerve.

## Article Information

### Conflicts of Interest

none

### Acknowledgement

The authors thank Dr Rui Kamada (Sapporo Kojinkai Memorial Hospital) for his valuable comments and suggestions on this case.

### Author Contributions

Azusa Shimabukuro: investigation, writing―original draft. Tadao Aikawa: conceptualization, investigation, writing―review and editing. Kenichi Iijima: investigation, writing―review and editing. Tohru Minamino: supervision. All authors have read and critically revised the manuscript and approved the final manuscript.

### Approval by Institutional Review Board (IRB)

Institutional Review Board approval is not required for case reports at our institution (Juntendo University Hospital, Tokyo, Japan). Therefore, no IRB approval code has been assigned to this case report.

### Consent for Publication

The authors confirm that written consent for submission and publication of this case report including images and associated text has been obtained from the patient in line with CARE guidelines.

### Permission to Reproduce Material from Other Sources

N/A

### Clinical Trial Registration

N/A
